# A Systematic Review of the Epidemiology of Mansonelliasis

**DOI:** 10.4314/ajid.v4i1.55085

**Published:** 2010

**Authors:** Barbara L Downes, Kathryn H Jacobsen

**Affiliations:** Department of Global & Community Health at George Mason University (Fairfax, Virginia, USA)

**Keywords:** mansonelliasis, filariasis, mansonella, epidemiology

## Abstract

Although infection with any of the three types of *Mansonella* species that affect humans is often asymptomatic, a large portion of the world's population is at risk of this vectorborne filarial nematode infection. No previous global review of the epidemiology of mansonelliasis has been conducted. A systematic review of the literature was conducted. Original research articles that provided population-based mansonelliasis prevalence rates were identified by searching the PubMed database using pre-defined eligibility criteria. Data from each of the forty-six included studies were extracted and compared. Mansonelliasis is a common infection in some parts of west and central Africa and Latin America, with significant variation in prevalence rates over small geographic spaces. The risk of infection increases with age and may be higher in males than females. Despite many similarities, the three agents that cause mansonelliasis have distinct biological, clinical, and epidemiological characteristics. Knowledge about mansonelliasis is important for making differential diagnoses, identifying the possible risks of co-infection with multiple filariases, and addressing the concerns of at-risk populations.

## Introduction

Mansonelliasis is one of several filarial nematode infections for which humans are the definitive host. This puts it in the same category as several parasitic infections of importance to global public health, including onchocerciasis, lymphatic filariasis, dracunculiasis, and loiasis. The three agents that cause mansonelliasis - *Mansonella perstans, M. streptocerca*, and *M. ozzardi* - vary in features such as anatomy and periodicity, the vectors that transmit the agent to humans, the clinical signs and symptoms they cause, and the world regions where they are endemic. While some of these major filarial infections have garnered international attention - onchocerciasis (river blindness) (Gardon et al., 1997) and dracunculiasis (Guinea worm) (Barry, 2007; Cairncross et al., 2002) have been the focus of global eradication efforts - mansonelliasis has been neglected.

This paper is the first systematic global review of the epidemiologic literature on all three forms of mansonelliasis. A systematic search strategy was used to identify 46 original scientific articles of the prevalence of mansonelliasis. These publications report on studies from 18 countries in Africa and Latin America. After providing a brief background on the key features of each of the three types of mansonelliasis, this paper provides a comparison of the epidemiology of these infections, with an emphasis on at-risk populations and geographic regions. Up-to-date epidemiological information is essential for making differential diagnoses, planning public health interventions, and advancing research in the field.

## Background on Mansonelliasis

### Agent and Vector Characteristics

Three types of *Mansonella*, which are filarial nematodes (roundworms), are known to infect humans: *M. perstans* (formerly *Dipetalonema perstans*), *M. streptocerca* (formerly *Dipetalonema streptocerca*), and *M. ozzardi* (CDC, 2008; Garcia, 2007; Heymann, 2004). The life cycles for all three species are similar, involving development in both an insect vector and a primate host. *Culicoides* (biting midges) are effective vectors for all three species; *Simulium* (black flies) are a vector only for *M. ozzardi* (Shelley, 2001). Both vectors require blood meals in order for their eggs to mature (Black et al., 2004). When a female arthropod takes a blood meal from an infected host, microfilariae are ingested by the insect, penetrate the insect's gut and go through several maturation stages in the thoracic muscles over 6 to 12 days before migrating to the head and proboscis, where they can be transferred to a primate through an insect bite (Black et al., 2004; CDC, 2008). Humans are the only known vertebrate host for *M. ozzardi*; other primates can serve as host to *M. perstans* and *M. streptocerca* (Garcia, 2007). After the vector deposits filarial larvae onto the skin of the host, the larvae penetrate into the bite wound, mature into adult worms, and then the adult female worms produce unsheathed microfilariae that circulate in the blood (all three species) or diffuse into the skin (*M. streptocerca* only) of the primate host (Black, 2004; CDC, 2008; Garcia, 2007). All three species have non-periodic microfilariae that circulate in peripheral blood throughout the day and night (Garcia, 2007; Mommers et al., 1994; Service, 2004). The size of the adult worms varies by species, and microfilariae differ in the shape of the tail and the distribution of body nuclei. Key differences between these species are highlighted on Table 1.

**Table 1:** Agent characteristics [CDC, 2004; Garcia, 2007; Heymann, 2004].

Agent	*Mansonella perstans*	*Mansonella streptocerca*	*Mansonella ozzardi*
Adult Size	4–8 cm × 0.06 mm	2 cm × 0.01 mm	3–5 cm × 0.07–0.15 mm
Microfilarial Characteristics	100–200 µm × 5 µm; blunt rounded tail; body nuclei extend to tip of tail	180–240 µm × 2.5–5 µm; curved hooked “Shepherd's crook” tail; body nuclei extend to tip of tail	170–240 µm × 3–4 µm; long thin pointed tail; body nuclei do not extend to tip of tail
Vector	*Culicoides* spp. (biting midges)	*Culicoides* spp. (biting midges)	*Culicoides* spp. (biting midges) and *Simulium* spp. (blackflies)
Hosts	humans, gorillas, and monkeys	humans and monkeys	humans
Signs / Symptoms	usually asymptomatic	often asymptomatic; may cause chronic pruritus (itchiness) and thick papules on skin	often asymptomatic; may cause malaise
Common Adult Locations	body cavities	subcutaneous tissues	subcutaneous tissues
Common Microfilarial Locations	blood	skin	blood
Diagnosis	peripheral blood smear	skin snip	blood smear
Recommended Treatment	mebendazole	DEC (diethylcarbamazine) / ivermectin	ivermectin
Geographic Range	Africa and the Americas	West and central Africa	the Americas

### Clinical Characteristics

Table 1 highlights key differences in signs and symptoms, diagnosis, and treatment between the three species. Infection with any of the three is often asymptomatic. Symptoms that do occur are related to the preferred location of the agent: *M. perstans* are typically found in body cavities, *M. streptocerca* in dermal and subcutaneous tissue, and *M. ozzardi* in subcutaneous tissues (Garcia, 2007; Heymann, 2004). Symptoms of infection with *M. perstans* may include pectoral and chest pains, periodic dizziness, joint and back pain, and ocular symptoms (Anosike et al., 2005b; Bregani et al., 2006; Bregani et al., 2007). Infection with *M. streptocerca*, which is found under the skin, is associated with cutaenous edema (build-up of fluid in the skin), thickening of the skin, formation of hypopigmented macules (flat blotches) and papules (raised bumps), and pruritus (itchiness) (Heymann, 2004; Fischer et al., 1997). *M. ozzardi* may cause symptoms that include skin rashes, headaches, fever, pruritus, lymphedema (swelling of the arms or legs), and joint pain (CDC, 2008; Garcia, 2007).

### Diagnosis and Treatment

Diagnosis and treatment also vary by species (Table 1). Blood smears that look for microfilariae are the easiest way to diagnose *M. perstans* and *M. ozzardi* (CDC, 2008). *M. streptocerca* microfilariae do not circulate in the blood, so it is necessary to take a skin snip (CDC, 2008). Care must be taken to differentiate mansonelliasis from onchocerciasis or other filarial infections (Fischer et al., 1997). Treatment must be specific to the infective agent. *M. perstans* is most effectively treated with mebendazole; ivermectin is not effective against *M. perstans*, but is the drug of choice for treating *M. ozzardi* (Garcia, 2007; Heymann, 2004). Both diethylcarbamazine (DEC) and ivermectin have been used to treat *M. streptocerca* infection (Garcia, 2007).

## Methods

Systematic reviews of the literature minimize the selection bias that may occur in narrative reviews that select articles by hand rather than by using a strict set of inclusion criteria. This methodical approach yields a valid and comparable set of research articles which together can reveal trends and gaps in the published research literature.

**Figure 1:**
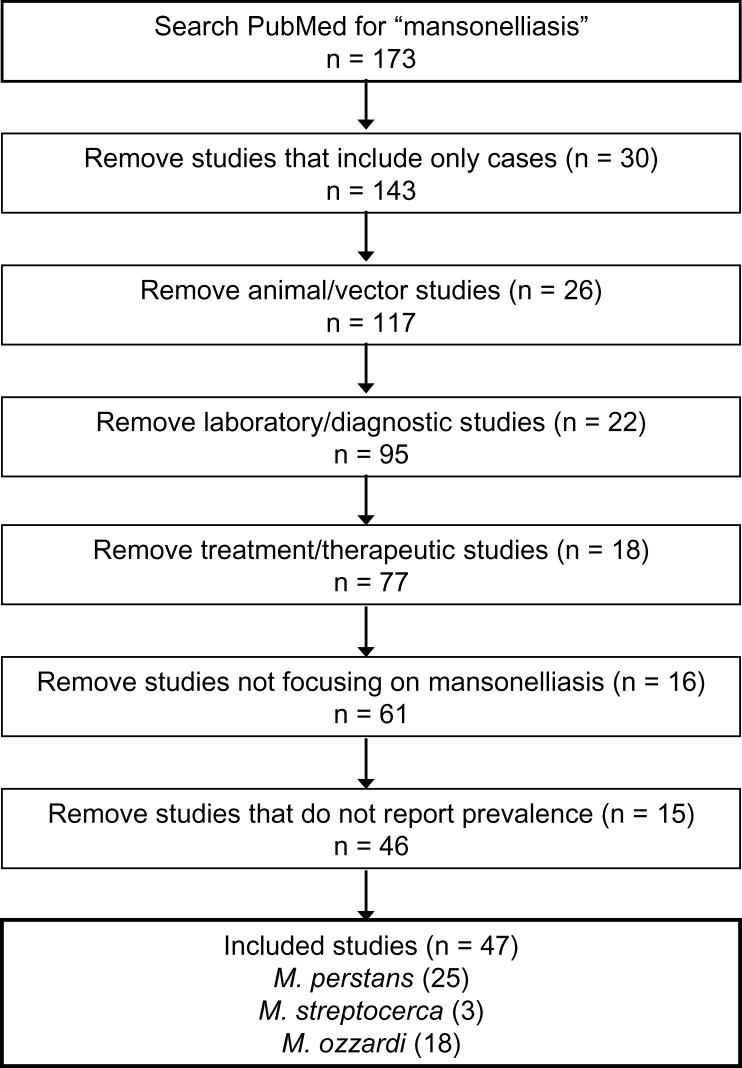
Search strategy.

A systematic review of original research articles focusing on the prevalence of mansonelliasis was conducted using PubMed, a database from the U.S. National Institutes of Health that searches all MEDLINE citations along with several other databases and older publications (Figure 1). A search for “mansonelliasis” yielded 173 results. The abstracts and/or full-texts of these articles were screened for eligibility. Of the 173 articles, 127 were ineligible: 30 that included only individuals with mansonelliasis and did not provide any population-based statistics, 26 that examined the vectors of infection rather than the human hosts, 22 that reported solely on laboratory techniques and diagnostic methods, 18 that evaluated treatment for mansonelliasis, 16 that focused on a disease other than mansonelliasis and only mentioned mansonelliasis in the commentary, and 15 additional articles that did not report population-based prevalence rates.

All of the 46 remaining articles were located and read, and information about the study country, study years, sample size, age range of participants, and prevalence was recorded. All languages were eligible for inclusion, and the 46 eligible articles were in English (39), Spanish (3), Portuguese (3), and French (1).

## Results

The goals of the systematic review were to identify the areas of the world where mansonelliasis has been studied, to identify the prevalence rate in affected communities, and to list the risk factors that have been identified for each species. These findings are presented below and in Tables 2, 3, and 4.

**Table 2:** Epidemiological studies of *M. perstans*.

Country	Study Year	Sample Size	Age Range (years)	Prevalence	Reference
Colombia	--	604	--	6%	Kozek, 1983
Burkina Faso	2001	3303	≥ 1	6%	Kyelem, 2003
Cameroon	1992	466	≥ 0.5	27%	Mommers, 1994
	--	1458	≥ 15	70%	Wanji, 2003
Congo	1985–1986	2313	≥ 1	29%	Noireau, 1989
Gabon	1984–1985	411	9–70	49%	Van Hoegaerden, 1987
Guinea	1989	829	≥ 10	66%	Vila Montlleo, 1990
Mali	--	40	18–65	75%	Keiser, 2003
Nigeria	2003–2004	373	--	3 %	Anosike, 2005a
	1996–2000	755	≥ 5	9%	Anosike, 2005b
	1997–1998	373	4–55	3%	Agbolade, 2001
	1988–1991	4183	0–70	29%	Anosike, 1992
	1993	840	≥ 1	15%	Useh, 1995
	1989	2552	--	11%	Akogun, 1992
	1984–1987	940	3–80	8%	Ufomadu, 1990
	--	845	--	13%	Udonsi, 1988
	1983–1984	1674	≥ 1	47%	Arene, 1986
	--	1351	≥ 1	46%	Udonsi, 1986
Sierra Leone	1993	630	5–70	6 %	Gbakima, 1996
Togo	--	182	--	42%	Schulz-Key, 1993
Uganda	2005–2006	1566	≥ 1	65%	Asio, 2009
	2003–2005	2499	14–47	21%	Hillier, 2008
	2003	12207	5–19	61%	Onapa, 2005
	1998	3548	--		Onapa, 2001
	1994–1995	233	≥ 14	96%	Fischer, 1997a
	1991–1993	1543	≥ 14	49%	Fischer, 1996

--: information not provided in article

**Table 3:** Epidemiological studies of *M. streptocerca*.

Country	Study Year	Sample Size	Age Range (years)	Prevalence	Reference
Central African Republic	--	267	1–100	14%	Okelo, 1988
Nigeria	1990–1992	1349	0–70	0.5%	Anosike, 1994
Uganda	1994–1995	806	≥ 14	61%	Fischer, 1997a

--: information not provided in article

**Table 4:** Epidemiological studies of *M. ozzardi*.

Country	Study Year	Sample Size	Age Range (years)	Prevalence	Reference
Bolivia	1997	594	0–85	26%	Bartoloni, 1999
Brazil	2007	129	≥ 2	30%	Mederios, 2008
	2006	543	--	19%	Cohen, 2008
	--	496	--	28%	Garrido, 2000
	--	386	--	4%	Shelley, 1975
	--	262	--	27%	Lage, 1964
Colombia	--	347	8–70	49%	Lightner, 1980
	--	627	--	3%	Kozek, 1984
	--	604	--	13%	Kozek, 1983
Haiti	--	1165	all	16%	Raccurt, 1980
Mexico	1956	329	--	61%	Biagi, 1956
Trinidad	--	4,488	≥ 5	5%	Nathan, 1979
Venezuela	--	1057	--	10%	Gomez, 2000
	1983–1989	423	--	36%	Medrano, 1992
	--	139	--	58%	Godoy, 1980
	1977	146	≥ 6	22%	Le Bras, 1978
	--	187	--	10%	Beaver, 1976

--: information not provided in article

*Mansonella perstans* is found in both Africa and the Americas, but has primarily been studied in Africa (Table 2). The prevalence in endemic areas varies greatly even within small geographic regions. For example, a 2003 study of school children in Uganda showed variation in school-level prevalence ranging from 0.4% to 72.8% (Onapa et al., 2005), and a 2005–2006 study in Uganda found a rate of 57.7% in one community and 76.5% in a neighboring community (Asio et al., 2009). Other studies from Uganda have found village prevalence rates as low as 2% (Onapa et al., 2001) and 21% (Hillier et al., 2008) and as high as 96% (Fischer et al., 1997). A study in Cameroon found village prevalence rates ranging from 55% to 100% (Wanji et al., 2003), while another study from Cameroon found a lower prevalence rate of 26.6% (Mommers et al., 1994). A study of villages in Congo found village rates ranging from 22.0% to 89.5% (Noireau et al., 1989) and a study in Burkina Faso found village rates ranging from 3.5% to 14% (Kyelem et al., 2003). Prevalence rates from other studies in West and Central Africa demonstrate a similarly wide infection rate, ranging from 3.2% to 47% in Nigeria (Agbolade and Akinboye, 2001; Akogun, 1992; Anosike et al., 1992, 2005b; Arene and Atu, 1986; Udonsi, 1986, 1988; Ufomadu et al., 1991; Useh and Ejezie, 1995) and 6.0% in Sierra Leone (Gbakima and Sahr, 1996) to 49.1% in Gabon (Van Hoegaerden et al., 1987), 66.3% in Guinea (Vila Montlleo, 1990), and 75% in Mali (Keiser et al., 2003). The only recent study from Latin America was conducted among an indigenous population in Venezuela and found a prevalence of 11.3% (Gómez and Guerrero, 2000). A study from Colombia found a prevalence of 6% in affected communities in the 1980s (Kozek et al., 1983).

Co-infection with *M. perstans* and other filarial infections appears to be common. 42.3% of onchocerciasis patients in a study in Togo were co-infected with *M. perstans* (Schulz-Key et al., 1993), 36.9% of participants in a study in Cameroon were infected with both *M. perstans* and *O. volvulus* (Wanji et al., 2003), 14% of participants in a study in Gabon had both *M. perstans* and *L. loa* (Van Hoegaerden et al., 1987), 10.1% of persons with *M. perstans* infection in a study from Nigeria also had *L. loa* (Ufomadu et al., 1991), and 9% of participants in a study conducted in the Congo were infected with both *M. perstans* and *L. loa* and 7% had both *M. perstans* and *M. streptocerca* (Noireau et al., 1989). Given the concern that has been raised about filarial co-infection with other agents, this may be an area of concern (Boussinesq et al., 2003; Gardon et al., 1997).

Most studies that examined differences in *M. perstans* prevalence by sex found no difference between males and females (Agbolade et al., 2001; Asio et al., 2009; Boussinesq et al., 2003; Gbakima and Sahr, 1996; Ufomadu et al., 1991; Useh et al., 1995) although several other studies observed a higher rate in males than females (Anosike et al., 2005b; Mommers et al., 1994; Noireau et al., 1989; Wanji et al., 2003). Studies of the association between age and infection consistently found a higher rate in adults than children (Agbolade et al., 2001; Anosike et al., 2005b; Asio et al., 2009; Gbakima and Sahr, 1996; Keiser et al., 2003; Mommers et al., 1994; Noireau et al., 1989; Ufomadu et al., 1991; Wanji et al., 2003).

*Mansonella streptocerca* occurs in west and central Africa, and has been the focus of relatively few studies (Table 3). As was found for *M. perstans*, the prevalence rate appears to vary widely within endemic areas. A study in western Uganda in the mid-1990s found that the village prevalence ranged from 5% to 89% (Fischer et al., 1997). A study from the 1980s conducted in the Central African Republic found a prevalence of 13.5% (Okelo et al., 1988) and a study in Nigeria from the early 1990s found a prevalence of 0.5% (Anosike and Onwuliri, 1994). Additional studies are required to establish the geographic range where this agent is endemic and to identify risk factors.

*Mansonella ozzardi* infection, also known as mansonellosis, occurs only in the Americas (Table 4). In the past ten years, the results of cross-sectional studies from Brazil (Cohen et al., 2008; Garrido and Campos, 2000; Medeiros et al., 2008), Bolivia (Bartoloni et al., 1999), and Venezuela (Gómez and Guerrero, 2000) have been published. Most of the studies in Brazil and Venezuela were conducted in communities located along rivers in the Amazon basin and focused on indigenous groups. The prevalence rates ranged from 9.9% (Gómez and Guerrero, 2000) to 18.9% (Cohen et al., 2008) to 28.2 % (Garrido and Campos, 2000) to 30.2% (Medeiros et al., 2008). Older studies from Brazil found prevalence rates ranging from 4% (Shelley, 1975) to 27% (Lage, 1964). The Bolivian study also focused primarily on an indigenous population, and found a total prevalence of 0.7% in one town and 26% in a neighboring town of 26% (Bartoloni et al., 1999), which suggests the same diverse range of prevalence rates found for the other species. Prevalence rates from studies of rural areas in Venezuela ranged from 11% (Beaver et al., 1976) to 22% (Le Bras et al., 1978) to 30% (Formica and Botto, 1990) to 36% (Medrano et al., 1992) to 58% (Godoy et al., 1980). In a study from the 1970s, about 16% (Raccurt et al., 1980) of inhabitants surveyed from Bayeux, Haiti, were found to be infected with *Mansonella ozzardi*. In Colombia, prevalence rates ranged from 3% (Kozek et al., 1984) to 13% (Kozek et al., 1983) to 49% (Lightner et al., 1980). These studies consistently found that risk of infection increased with age (Bartoloni et al., 1999; Le Bras et al., 1978; Medeiros et al., 2008; Nathan et al., 1979). Although one study from Trinidad in the 1970s indicated an increased risk of infection in males (Nathan et al., 1979), more recent studies from Bolivia (Bartoloni et al., 1999) and Brazil (Medeiros et al., 2008) found no differences in prevalence by sex. Thus, aside from age no risk factors have been firmly established.

## Discussion

While the three agents that cause mansonelliasis share these similarities, they are distinct infections with unique agent, clinical, and epidemiological characteristics. Although infection is usually asymptomatic, millions of people worldwide - especially those in rural areas - are at risk. This systematic review shows that mansonelliasis may be a common infection in parts of Latin American and west and central Africa, with significant variation in prevalence rates over small geographic spaces, but the review also highlights the lack of current information about the prevalence of mansonelliasis in most areas likely to at risk. Also, although the review indicates that the risk of infection increases with age and may be higher in males than females, there is a need for additional work to identify specific demographic and environmental risk factors. Updated information will be important for making differential diagnoses in endemic and epidemic areas, promoting measures to control vectors in areas with significant burden from the disease, identifying the possible risks of co-infection with multiple filariases, and addressing the concerns of at-risk populations.
